# Microbial Diversity and Activity of Biofilms from Geothermal Springs in Croatia

**DOI:** 10.1007/s00248-023-02239-1

**Published:** 2023-05-20

**Authors:** Ema Kostešić, Maja Mitrović, Katarina Kajan, Tamara Marković, Bela Hausmann, Sandi Orlić, Petra Pjevac

**Affiliations:** 1 Division of Materials Chemistry, Ruđer Bošković Institute, Zagreb, Croatia; 2 Center of Excellence for Science and Technology-Integration of Mediterranean Region (STIM), Split, Croatia; 3 Croatian Geological Survey, Zagreb, Croatia; 4 Joint Microbiome Facility of the Medical University of Vienna and the University of Vienna, Vienna, Austria; 5 Department of Laboratory Medicine, Medical University of Vienna, Vienna, Austria; 6 Centre for Microbiology and Environmental Systems Science, Department of Microbiology and Ecosystem Science, University of Vienna, Vienna, Austria

**Keywords:** Hot spring, Biofilm, 16S rRNA gene amplicon analysis, BONCAT, CARD-FISH

## Abstract

Hot spring biofilms are stable, highly complex microbial structures. They form at dynamic redox and light gradients and are composed of microorganisms adapted to the extreme temperatures and fluctuating geochemical conditions of geothermal environments. In Croatia, a large number of poorly investigated geothermal springs host biofilm communities. Here, we investigated the microbial community composition of biofilms collected over several seasons at 12 geothermal springs and wells. We found biofilm microbial communities to be temporally stable and highly dominated by *Cyanobacteria* in all but one high-temperature sampling site (Bizovac well). Of the physiochemical parameters recorded, temperature had the strongest influence on biofilm microbial community composition. Besides *Cyanobacteria*, the biofilms were mainly inhabited by *Chloroflexota*, *Gammaproteobacteria*, and *Bacteroidota*. In a series of incubations with *Cyanobacteria*-dominated biofilms from Tuhelj spring and *Chloroflexota*- and *Pseudomonadota*-dominated biofilms from Bizovac well, we stimulated either chemoorganotrophic or chemolithotrophic community members, to determine the fraction of microorganisms dependent on organic carbon (in situ predominantly produced via photosynthesis) versus energy derived from geochemical redox gradients (here simulated by addition of thiosulfate). We found surprisingly similar levels of activity in response to all substrates in these two distinct biofilm communities, and observed microbial community composition and hot spring geochemistry to be poor predictors of microbial activity in the study systems.

**Supplementary Information:**

The online version contains supplementary material available at 10.1007/s00248-023-02239-1.

## Introduction

Geothermal aquifers are permeable layers of fluid-bearing rock that store some of the heat from the Earth’s interior [1]. Their high permeability allows continuous circulation of heat and fluids, but also the formation of temperature, chemical, and redox gradients [2, 3]. In consequence, hot springs function as autonomous bioreactors that allow colonization by diverse, specially adapted microbial communities (e.g., [4, 5]). These communities are often dominated by thermophilic Archaea and Bacteria that can survive in a temperature range of 50–121°C [6–9]. Extreme temperatures and pH have been shown to contribute most to limiting microbial diversity in geothermal aquifers and hot springs (e.g., [10–13]), although other environmental factors, such as light quality [14] and spring geochemistry [15–17], have also been shown to affect microbial community composition. Adaptation to the extreme conditions in hot springs requires genomic plasticity and metabolic flexibility of microorganisms, making them good candidates for the discovery of bioactive molecules of industrial and biotechnological interest [8, 18]. A well-known example is the discovery of *Thermus aquaticus* by T. Brock in the hot springs of Yellowstone National Park [19] and its DNA polymerase implementation in the polymerase chain reaction [20], which rapidly increased the interest in the study of microorganisms from geothermal springs.

Thermal springs are composed of various microenvironments such as sediments, water columns, microbial mats, and biofilms [21]. Biofilms are defined as associations of microbial cells, either of a single or multiple species, that have formed a community in an aggregated manner, attaching themselves to each other or to a surface by secreting extracellular polymeric substances (EPS) [22–24]. There is extensive literature describing the development of biofilms and the different phases of their establishment [24–30]. Hot spring biofilms have also been studied for decades and interest in them has still not waned. They are accessible, relatively stable, high biomass systems that harbor an enormous biotechnological potential [8, 31, 32]. Their composition is usually simple enough to be evaluated by traditional methods such as microscopy [33], but as they form on redox interfaces with well-defined environmental gradients, they can host a wide variety of metabolically diverse microorganisms (e.g., [34–36]). Although a large portion of biofilm-dwelling microorganisms can be isolated into pure culture, activities in pure cultures do not necessarily reflect in situ activities and do not provide insight into interactions within the biofilm community [37]. In summation, despite the comparatively low species richness, it is not trivial to understand the drivers behind the observed community structure and the biogeochemical cycling mechanisms due to the complex microbial interaction networks that form in hot spring biofilms [38].

The body of literature on the microbial community composition and the genomic landscape of hot spring biofilms continues to grow. However, despite the diverse suit of available methods to investigate the ecology and ecophysiology of biofilm microbial communities, such data are scarce for hot spring biofilms. In microbial ecology, incorporation of radio or stable isotope-labeled, or analogous substrates into microbial biomass is often used to trace metabolic activity [39]. Such substrate tracing approaches have been used to determine bulk activities of mixed biofilm communities (e.g., [21]) and to link specific metabolic activities to certain groups of hot spring biofilm organisms (e.g., via lipid biomarkers, [35, 40]). The same principle can also be used to link the utilization of a substrate to an individual cell via so-called single cell approaches [41–43]. Microbial cells that form hot spring (and other) biofilms are enclosed in an EPS matrix that restricts the diffusion of large molecules, and hinders the separation of cells and matrix components often necessary for downstream analysis, making the application of some physiology-targeted single-cell methods and analyses challenging [44]. Nevertheless, there is a plethora of studies investigating biofilm communities at single cell resolution (e.g., [45–47]). It is thus surprising that single-cell ecophysiological methods have almost never been applied to geothermal biofilms: only a few microautoradiography (MAR) experiments conducted on photosynthetic hot spring biofilms and mats from different pools in Yellowstone have been published to date [48–51].

MAR visualizes the incorporation of radiolabeled isotopes into microbial cells and can be combined with fluorescence in situ hybridization (FISH) to identify active microorganisms, but sample processing is time consuming and requires specialized laboratory equipment and radioactive waste management [39, 42, 52]. Nanoscale secondary ion mass spectrometry (nanoSIMS) enables the visualization of stable isotope incorporation, circumventing the waste and safety issues related to working with radioisotopes, but low throughput and the need for sophisticated instrumentation limit its application in practice [39, 42, 52]. In addition, nanoSIMS only examines the surface of a sample, and although tomography is possible [53, 54], it is not practical for biofilms. Notably, both MAR and nanoSIMS are destructive methods that prevent downstream analyses, such as genome sequencing or cultivation [42]. Bioorthogonal non-canonical amino acid tagging (BONCAT), which can be used to visualize a large diversity of translationally active cells and can reveal under which conditions cells and cell populations are active [55, 56], has fewer drawbacks than other currently available single-cell methods. BONCAT is based on the incorporation of azide- or alkyne-group containing l-methionine analogs, whereupon newly synthesized proteins can be detected using azide-alkyne click chemistry [57]. BONCAT requires a relatively simple infrastructure available to most laboratories and is a rapid and nondestructive method compatible with many other approaches such as (meta)genomics, (meta)proteomics, nanoSIMS, fluorescence activated cell sorting (FACS), and FISH [39, 42, 58–60]. The combination of BONCAT with FISH allows to directly link the identity and activity of microorganisms in environmental samples by fluorescence microscopy [45]. Albeit being a relatively novel method, it has already been successfully applied on various environmental sample types, including biofilms [45, 52, 56, 59, 61].

**Fig. 1:**
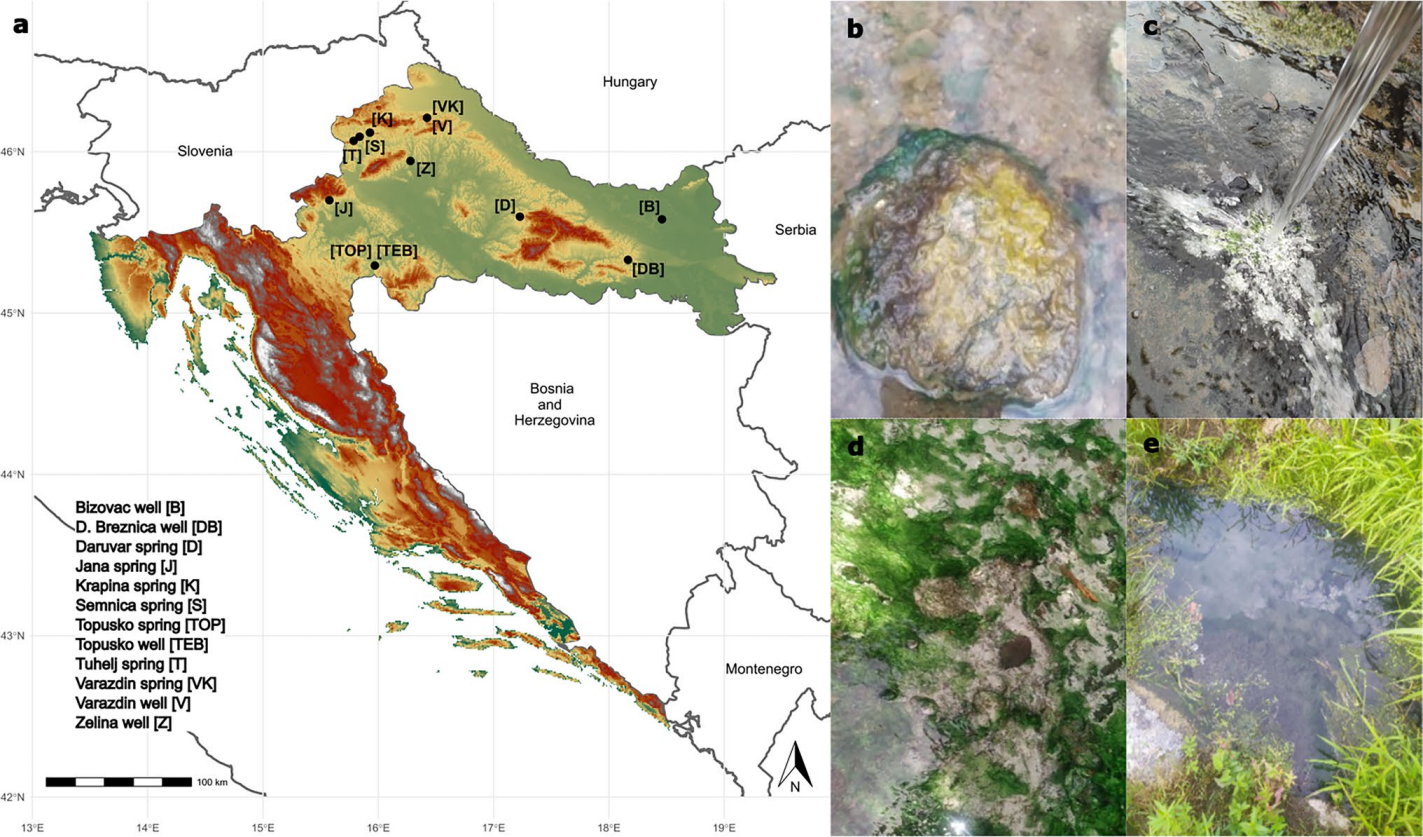
**a** Locations of thermal sites with investigated biofilms. **b** Close-up of different colored biofilms formed in the Topusko spring. **c** Biofilms formed in D. Breznica well. **d** Close-up of green and white biofilms formed in Tuhelj spring. **e** Tuhelj spring

In this study, we applied a combination of catalyzed reported deposition (CARD) FISH and BONCAT to investigate the identity and activity of microorganisms inhabiting two contrasting hot spring biofilms, supplied with substrates stimulating either organotrophic or lithotrophic microbial activity. First, we applied 16S rRNA gene amplicon sequencing to screen the microbial community composition in biofilms collected from geothermally active sites in northern and northeastern Croatia (Fig. 1). This region, characterized by a high average geothermal gradient (49°C/km), surface heat flow (76 mW/m^2^), and shallow Mohorovičić discontinuity, hosts a large number of geothermal sites [62, 63], many of which contain biofilms that have not been previously studied. In a recent study [63], we characterized the hydrochemistry of the geothermal springs and investigated the microbial community composition of geothermal waters. However, the environmental conditions of geothermal waters differ significantly from the microenvironment in biofilms, where nutrients are entrapped and microorganisms form metabolically and functionally integrated consortia [64, 65]. In consequence, distinct microbial communities can be expected to occur in geothermal biofilms. To gain insight into the diversity and metabolic activity of biofilm microorganisms in hot springs, we initially characterized the microbial community of biofilms collected from 12 geothermal sources over three sampling seasons. Subsequently, two distinctly colonized biofilms from geothermal sources with different geochemistry were supplied with substrates that stimulated either chemoorganotrophic or thiosulfate-dependent chemolithotrophic microbial activity. Although we were able to confirm the contribution of select microbial community members to these metabolic processes, the results of the incubation experiments did not fully align with hypothesized activities derived from microbial community composition and thermal water chemistry data. Our findings underline how difficult it is to correctly infer microbial functions from taxonomic data, even in relatively simple systems such as hot spring biofilms.

## Materials and methods

### Sampling and Physicochemical Measurements

Up to three samplings of biofilms from 12 geothermal sources located at 10 geothermally active sites in Croatia (Fig. 1a; Table S1) were conducted in autumn 2019, spring 2020, and autumn 2020, while additional biofilm samples from two sites (the Bizovac well and the Tuhelj spring) were collected in spring 2021 (Table S1). Depending on the presence and localization of biofilms, samples were collected in a depth range from the air–water interface to up to 0.5 m water depth, using a sterile spatula and tweezers. The biofilm samples were stored in sterile 50-mL tubes and submerged in the corresponding geothermal water. If multiple different colored biofilms were present at one site (e.g., Fig. 1b), they were sampled separately. Likewise, multilayered biofilms were separated according to the layer color and further processed separately. After sampling, electrical conductivity (EC), dissolved oxygen (O_2_) concentration, pH, and water temperature in geothermal spring waters were measured in situ using a WTW multiparameter probe (WTW, Germany) (Table S1). Sampling of biofilms and in situ measurements of psychochemical parameters in geothermal spring waters were conducted as close as possible to the spring source, and the measured parameters reflect the geochemistry experienced by the sampled biofilms. The concentrations of hydrogen sulfide (H_2_S) in spring waters directly adjacent to or above the biofilms were determined using Reagent sulfide 1 and Reagent sulfide 2 according to the USEPA Methylene Blue Method with a HACH DR3900 spectrometer, as specified in DR manual 1 (https://www.hach.com/asset-get.download-en.jsa?id=7639983731) (Table S1). If necessary, samples were diluted for H_2_S concentration measurements. For further chemical analysis in the laboratory, water samples were collected in 50-mL tubes and 500-mL plastic bottles. All samples were brought to the laboratory within a few hours after collection. Chemical analyses were performed at the Croatian Geological Institute (see [63] and Supplementary Material for further details). The concentrations of cations (sodium, potassium, calcium, ammonium, and magnesium) and anions (chloride, nitrate, and sulfate), alkalinity, SiO_2_ concentrations, and dissolved organic (DOC) and inorganic (DIC) carbon concentration were determined the same evening after returning from the field site (Table S1). The results of these chemical analyses were in parts already published previously in [63], where a detailed description of sampled geothermal sites can also be found.

### Biofilm DNA Extraction, 16S rRNA Gene Amplification, Sequencing, and Data Processing

After excess water was removed from the collected biofilm samples by decanting and pipetting, 0.5 g of each biofilm sample was weighed into beat beating tubes for DNA extraction. According to the manufacturer’s guidelines, total genomic DNA was extracted using the DNeasy PowerSoil Kit (Qiagen GmbH Hilden, Germany). The hypervariable V4 region of the 16S rRNA gene was performed using primer pairs 515F (5′-GTGYCAGCMGCCGCGGTAA-3′) [66] and 806R (5′-GGACTACNVGGGTWTCTAAT-3′) [67] at the Joint Microbiome Facility of the Medical University of Vienna and the University of Vienna (project IDs JMF-2007-4, JMF-2103-13, JMF-2110-16). Samples were amplified, barcoded, purified, normalized, and prepared for sequencing as previously described [68] and sequenced in pair-end mode (v3 chemistry, 2 × 300 bp) on an Illumina MiSeq instrument. Amplicon pools were extracted from raw sequencing data using default FASTQ workflow parameters (BaseSpace; Illumina), filtered for PhiX contamination using BBDuk (BBtools) [69, 70], and demultiplexed using the Python package demultiplex (Laros JFJ, github.com/jfjlaros/demultiplex), allowing for one mismatch per barcode and two mismatches for linker and primer sequences. FASTQ reads were trimmed at 220 and 150 nt, respectively, with an error of 2 allowed for forward and reverse reads each. Amplicon sequence variants (ASVs) were inferred in pooled mode in the DADA2 R package version 1.20.0 (R 4.1.1, [70, 71]) using standard settings, and classified against the SILVA reference database (SILVA release 138) using the SINA classifier, version 1.6.1 [72]. Prior to downstream analysis, ASVs classified as eukaryotes, mitochondria, or chloroplasts, as well as unclassified ASVs were removed from the dataset, as were singletons and doubletons. After filtering, samples with less than 3000 sequence reads were excluded from the dataset. The final ASV table contained 4201 ASVs and 59 samples with read counts greater than 3000. All statistical analyses were performed in the R environment (v. 4.2.0) using Bioconductor v3.15 packages TreeSummarizedExperiment [73], phyloseq [74], mia [75], and microViz [76] and visualized using the package ggplot2 [77]. The similarity of prokaryotic community between biofilm samples as a function of their environmental parameters was tested using non-metric multidimensional scaling (NMDS) based on Bray–Curtis dissimilarity distance. Permutational multivariate analysis of variance (PERMANOVA) was used to test the extent to which microbial communities were affected by sampling seasons, locations, selected environmental parameters, and biofilm color.

### Data Availability

16S rRNA gene amplicon sequencing data have been deposited in SRA (the Sequence Read Archive) under the BioProject accession number PRJNA889237.

### Incubation Experiments for Bizovac Well and Tuhelj Spring Biofilms

**Fig. 2:**
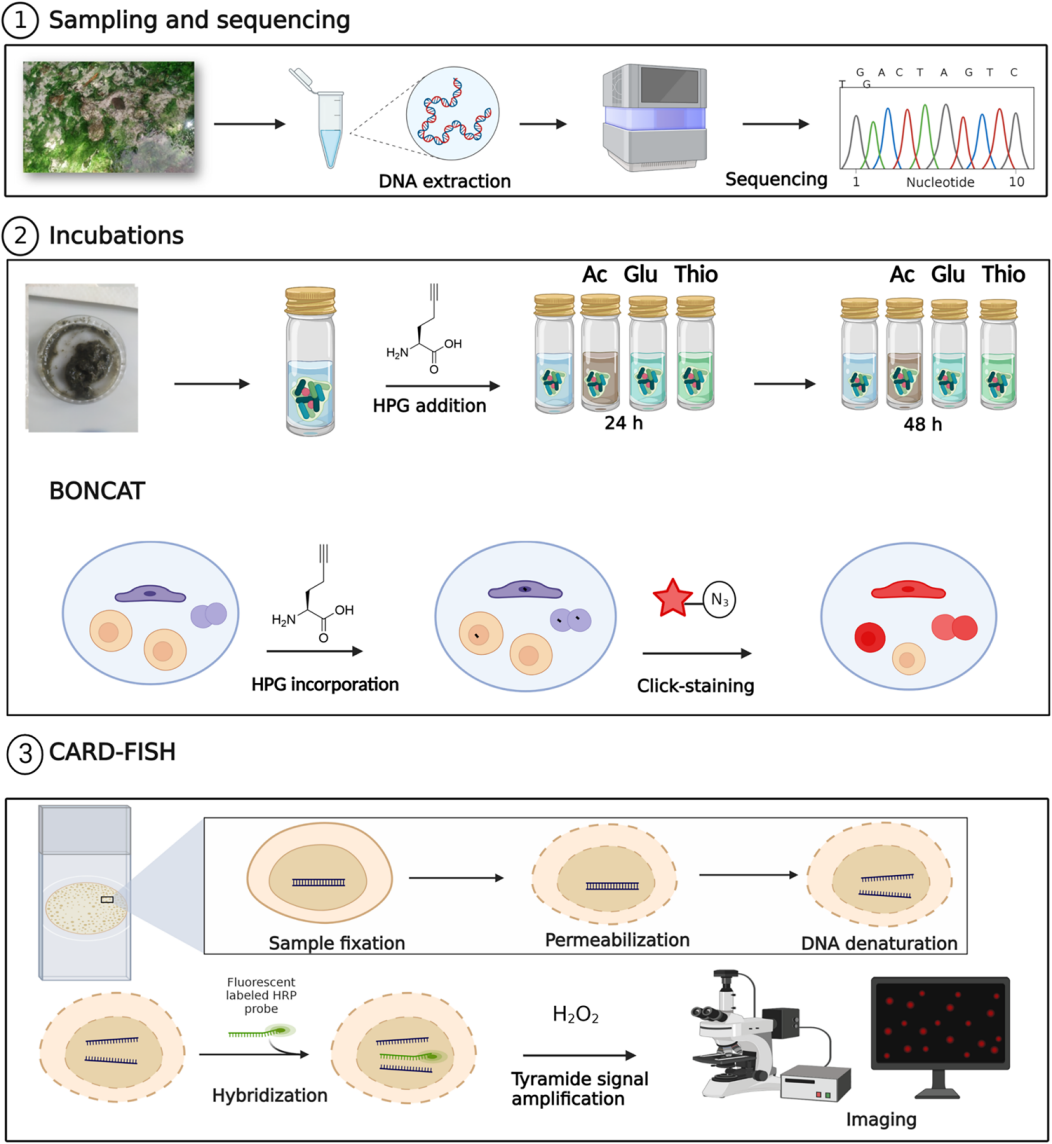
A schematic overview of the experimental workflow applied for hot spring biofilm characterization in this study: (1) characterization of the biofilm microbial community composition; (2) incubation with the l-methionine analogue HPG under different substrate amendment in the dark, for 24 h and 48 h and BONCAT visualization of active cells; (3) CARD-FISH hybridization of target populations, sample imaging and image analysis

**Fig. 3:**
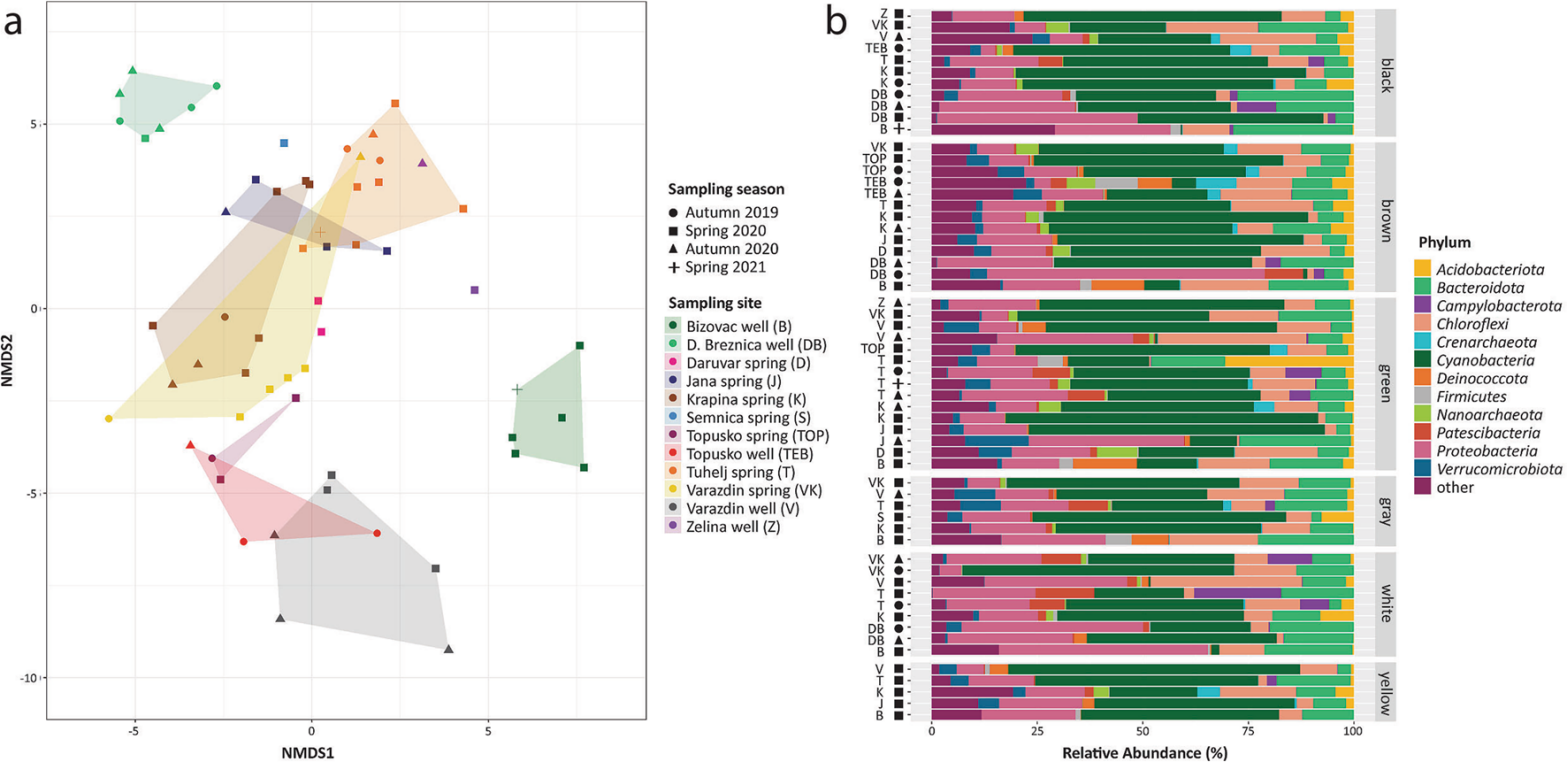
(**a**) NMDS clustering of hot spring biofilm microbial communities. Samples are colored by sampling location, while shapes represent the sampling season. (**b**) Microbial community composition clustered at phylum level. Phyla were cumulative relative abundance of ASVs exceeded 5% in at least one biofilm (sub)sample are displayed, while the residual community is clustered as “other.” Samples are grouped based on the color of biofilm (sub)samples. Sampling locations are indicated by the sample site abbreviations, while shapes represent the sampling season

Fresh biofilm samples were collected in spring 2021 from the Bizovac well, an atypical biofilm not dominated by *Cyanobacteria* from a moderately sulfidic, high-temperature geothermal source, and from the Tuhelj spring, representing a *Cyanobacteria*-dominated biofilm from a low-sulfide system (Fig. 3; Table S1). We characterized the biofilm microbial community of these freshly collected samples by 16S rRNA gene amplicon sequencing and confirmed that it resembles the composition of the previously analyzed biofilm samples collected in 2019 and 2020 from the respective source environment (Fig. 3a; Fig. S1). To assess the activity of microorganisms in hot spring biofilms sustained by different reducing equivalent sources, we designed a set of incubation experiments that stimulated either aerobic chemoorganotrophic or chemolithotrophic microbial activity. Since the redox gradients in the here investigated hot springs, including Bizovac well and Tuhelj spring, were partially characterized by the presence of sulfide-oxygen transitions zones ([63], Table S1), we provided thiosulfate as reducing equivalent source for chemolithotrophic microorganisms in our experiments. Unlike sulfide, thiosulfate is stable under oxic conditions and non-toxic at elevated concentrations, and the majority of sulfide oxidizers can also utilize thiosulfate [78]. Glucose or acetate amendments were used as organic substrates and reducing equivalents, as previous studies have shown that both glucose and acetate are released by *Cyanobacteria* as products of glycogen degradation under dark and anaerobic conditions [79, 80]. The combination of BONCAT and CARD-FISH was performed as described below (Fig. 2). For each incubation setup (Fig. 2), biofilm material (0.5 g wet weight) was suspended in 10 mL sterile filtered (0.2 μm pore size) corresponding geothermal spring water in glass vials and incubated for 48 h at source temperature. To monitor activity, l-homopropargylglycine (HPG) was added to the sample at a final concentration of 50 μmol L^−1^ alongside substrates (sodium acetate, glucose, and sodium thiosulfate) at a final concentration of 1 mmol L^−1^. Acetate and glucose solutions were prepared by dissolving the appropriate weight of substrate in autoclaved MQ water, while the thiosulfate media stock was prepared according to [81]. To assess native activity, a set of incubations was also performed without substrate amendment, with HPG addition only. All incubations were performed in the dark to minimize the effect of additional photosynthetically produced substrates on the experiment. After 24 and 48 h, samples were removed from the incubator and 2 mL of 0.5% Tween 20 (Promega) was added [82]. Samples were then macerated, vortexed at maximum speed for 5 min, sonicated at standard instrument settings for 1 min (Microson XL 2000) [83], and centrifuged at 500 × *g* for 5 min to separate the particles from the cell fraction [82]. The supernatant was sequentially filtered through 10 μm and 3 μm pore size polycarbonate filters to remove filamentous *Cyanobacteria*. The final filtrate was fixed with paraformaldehyde at a final concentration of 3% for 1 h, at room temperature, in the dark [83]. Samples were then filtered onto a 0.2 μm pore size filter, examined under the microscope using 4′,6-diamidino-2-phenylindole (DAPI) stain (10 μg μL^−1^), and the filters were stored at −20°C until the click reaction was performed.

BONCAT was performed according to [57]. Briefly, filter pieces were placed sequentially in 50, 80, and 96% EtOH for 3 min to dehydrate and permeabilize the cells. For the Cu(I)-catalyzed click reaction, a dye premix was prepared containing 1.25 μL of a 20 mmol L^−1^ CuSO_4_ solution, 1 μL of a 2.5 mmol L^−1^ Cy5-alkyne dye, and 2.5 μL of a 50 mmol L^−1^ tris-hydroxypropyltriazolylmethylamine chelating agent. While allowing the dye-premix to react, 12.5 μL of each 100 mmol L^−1^ sodium ascorbate and 100 mmol L^−1^ aminoguanidine hydrochloride were added to 221 μL phosphate buffered saline (PBS). After mixing the dye-premix with the rest of the buffer, the filters were added directly to the click mixture and incubated at room temperature in the dark for 1 h. The filters were then washed three times in PBS for 3 min each, dehydrated in 50% EtOH for 3 min, and stored at −20°C until the CARD-FISH procedure.

CARD-FISH was performed according to [84]. Briefly, after the filters thawed, they were dehydrated in a series of EtOH and embedded in 0.1% low melting point agarose. Permeabilization was performed with a 10 mg mL^−1^ lysozyme solution for 1 h and 60 U mL^−1^ achromopeptidase solution for 30 min, both at 37°C, followed by incubation in 0.1 mol L^−1^ HCl for 1 min at room temperature. Inactivation of endogenous peroxidases was achieved by 3% H_2_O_2_ treatment. Subsequently, 1.5 μL of a 50 ng μL^−1^ HRP-probe solution was added to 400 μL of hybridization buffer of corresponding stringency (Table S2). Filters were added directly to the solution and hybridized overnight at 46°C. To improve detection of microorganisms, the tyramide signal was amplified by catalyzed reporter deposition (CARD) [85]. Before tyramide signal amplification, washing was performed in washing buffer at 48°C for 10 min in a water bath and in Triton-X–PBS for 5 min. Filters were placed in a mixture of 1 mL of amplification buffer and 2 μL of tyramide solution (either Cy3 or Atto-488), incubated at 46°C for 20 min, and washed in Triton-X–PBS, MQ water, and absolute EtOH. After drying, filters were placed on the slide and embedded in Citifluor Vectashield DAPI (10 μg μL^−1^) solution. Oligonucleotide probes for CARD-FISH were selected based on 16S rRNA gene amplicon analysis of biofilms from previous seasons (Table S2). The NONEUB probe was used as a negative control for all hybridizations.

### Image Processing and Analysis

Images of the samples were acquired using an epifluorescence microscope (model DMi8; Leica, Germany) equipped with the Thunder Imaging System at a resolution of 2048 × 2048 pixels. For each sample, Z-stacks of 4–10 fields of view were acquired, in each channel individually as well as their overlay. The final images represent the maximum projection of all stacks. After inspection of the samples on the microscope, biovolume analysis was performed using *daime* software (v. 2_2_3) [86]. Images were processed with 2D filter histogram stretching to remove any background noise before automatic segmentation in ISODATA or Custom mode. After examining the results of various segmentation methods for each sample, the biovolume fractions of CARD-FISH positive cells/cell aggregates were determined against BONCAT positive ones, and vice versa. Results acquired through the *daime* software can be found in Table S2.

## Results

### Geothermal Spring Characteristic and Seasonal Biofilm Sampling

The here sampled geothermal springs and wells (*n* = 12) differed significantly in terms of their geochemistry (Table S1, [63]). In general, samples from the well Topusko had the highest temperature (TEB, 64.8°C) while samples from the D. Breznica well (DB, 12.9°C) had the lowest temperature. The pH ranged from 6.3 to 8.3, DIC from 52 to 390 mg L^−1^, and DOC from 0.05 to 35.5 mg L^−1^ (Table S1). The highest concentration of H_2_S was observed in spring 2020 in the DB well (18.8 mg L^−1^), while concentrations of SO_4_^2−^ ranged from 9.3 to 196.9 mg L^−1^ (Table S1). Samples from the Zelina and Bizovac well had the highest NH_4_^+^ concentration (9.03, 8.82 mg L^−1^). A more detailed description and characterization of sampling sites according to their environmental parameters can be found in [63].

Based on 16S rRNA gene amplicon sequencing, the microorganisms inhabiting 59 biofilm (sub)samples collected over three seasons were clustered into 4201 ASVs. NMDS analysis showed that the microbial community of the biofilms mainly clustered by spring or well of origin (Fig. 3a). This result was verified by PERMANOVA, confirming source spring as a significant factor affecting biofilm community composition (*R*^2^ = 0.42, *P* < 0.001). Among the measured environmental parameters, the community structure was best explained by temperature (*R*^2^ = 0.8, *P* < 0.001) and electric conductivity (which correlated to the concentrations of sodium, potassium, and chlorine ions) (*R*^2^ = 0.5, *P* < 0.001) (Table S3). The season of sampling (*R*^2^ = 0.06, *P* = 0.08) and the color of the biofilm (sub)samples (*R*^2^ = 0.08, *P* = 0.89) did not significantly correlate with observed community structure at the ASV level.

Overall, the biofilm communities were highly diverse, with ASVs affiliating with 52 bacterial and 10 archaeal phyla. While geothermal waters at the same sampling site were dominated by ASVs affiliating to *Crenarchaeota*, *Nanoarchaeota*, *Campylobacterota*, and various *Pseudomonadota* lineages [63], almost all here analyzed biofilm samples were dominated by *Cyanobacteria*. *Pseudomonadota*, *Chloroflexota*, and *Bacteroidota* also displayed high relative abundances in almost all biofilm samples (Fig. 3b). The relative abundance of Archaea was comparatively low in all samples (7 ± 3% on average). Unlike most samples, biofilms collected from the Bizovac well were not dominated by *Cyanobacteria* (Fig. 3b). Instead, *Pseudomonadota*, *Chloroflexota*, *Deinococcota*, and *Bacteroidota* accounted for large fractions of the community composition in biofilm samples from this site. At the family and genus level, prokaryotic community composition was highly site-specific (Fig. S1): *Leptolyngbyaceae* and *Nostocaceae* were the relatively most abundant cyanobacterial families in all biofilms, except for those collected at the highly sulfidic low temperature well DB, where *Trychonema*-related ASV (family *Phormidiaceae*) dominated the cyanobacterial community. In samples from Bizovac well, *Cyanobacteria* were not only relatively less abundant overall, but also distinct from cyanobacterial communities of the remaining biofilms, with ASVs predominantly affiliating with unicellular, colony-forming members of the genus *Gloeocapsa* (family *Chroococcidiopsaceae*; Fig. S1). In general, Bizovac well samples displayed a relatively high diversity, with few highly abundant individual genera and rather distinct community composition compared to samples from other geothermal sites (Fig. 3; Fig. S1). Among thiotrophic community members, unicellular gammaproteobacterial *Halothiobacillaceae* (genus *Thiofaba*) dominated biofilms from the Bizovac well during all three sampling seasons, whereas filamentous gammaproteobacterial population (family *Thiotrichaceae*, genus *Thiothrix*; Fig. S1) and ASVs related to the genus *Sulfurovum* (*Campylobacterota*; Fig. S1) were detected in the samples from all other sites.

The initial community of freshly collected (spring 2021) biofilms from the Bizovac well used for the activity experiments was again dominated by *Bacteroidota* (28.1%), *Pseudomonadota* (27.3%), and *Chloroflexota* (11.1%), while biofilms from the Tuhelj spring were again dominated by *Cyanobacteria* (41.5%), but *Pseudomonadota* (13.8%) and *Chloroflexota* (14.7%) were also relatively abundant (Fig. 3b; Fig. S1).

### Chemoorganotrophic versus Chemolithotrophic Microbial Activity of Biofilms from Bizovac Well and Tuhelj Spring

**Fig. 4:**
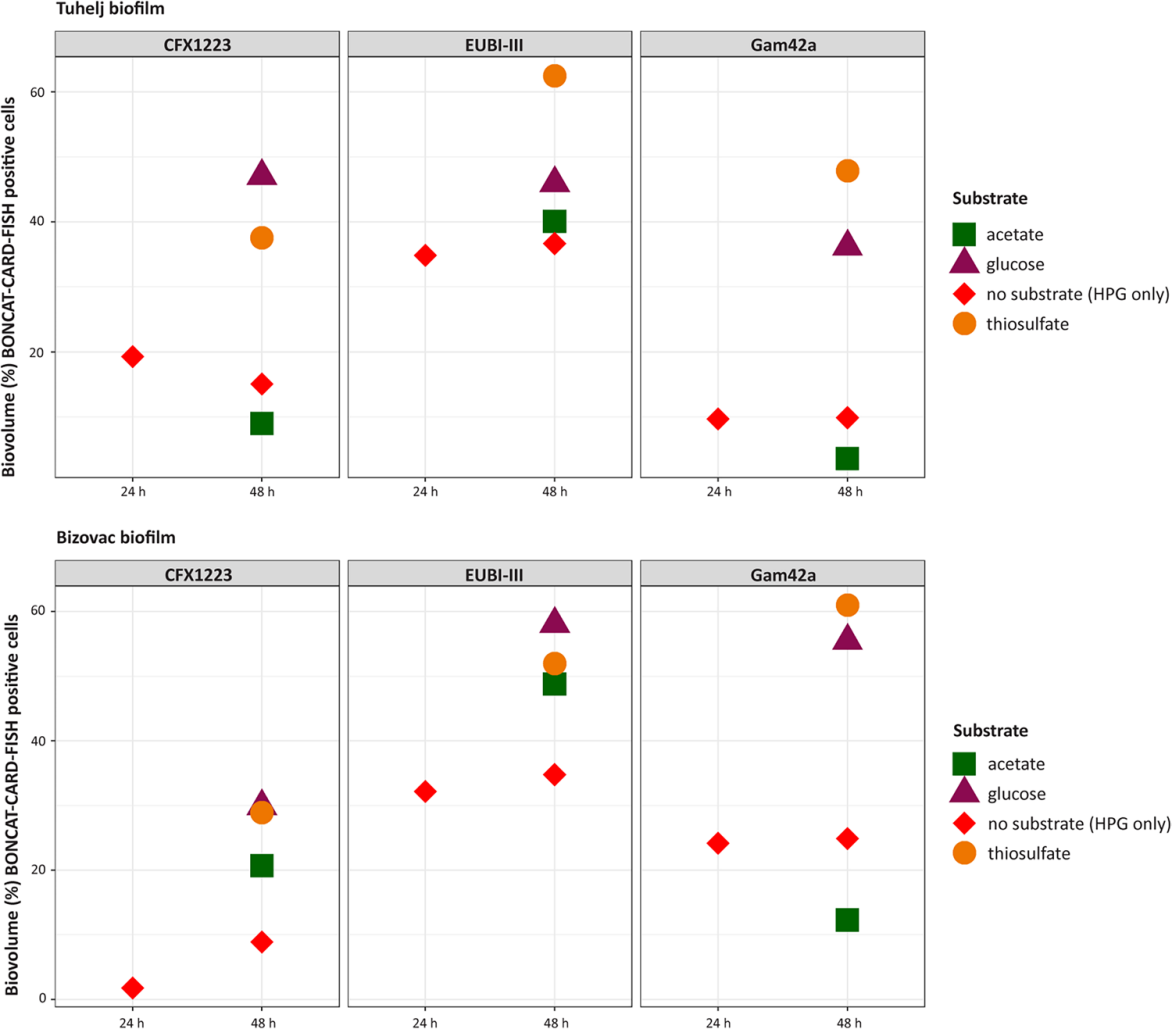
Biovolume of the active fraction (i.e., displaying BONCAT signal) of cells displaying a positive CARD-FISH single with probes targeting specifically the *Chloroflexota* (CFX1223), the *Gammaproteobacteria* (Gam42a), or all bacteria (EUBI-III) after 24 h without substrate amendment (HPG only), and 48 h without substrate amendment and in incubations amended with glucose, acetate or thiosulfate

As expected, the active fraction of the bacterial biofilm community (defined as displaying a CARD-FISH signal with the probe mixture EUBI-III targeting the majority of bacteria, and a positive BONCAT signal) was lowest when the biofilm samples were incubated without substrate amendment (i.e., HPG control). The active fraction of cells in the incubations without substrate amendment was very similar in both biofilm samples: after 24 h of incubation, 33% and 35% of EUBI-III positive cells displayed a BONCAT signal in Bizovac and Tuhelj biofilm incubations, respectively (Fig. 4). Furthermore, the fraction of active cells in substrate amendment free controls did not change significantly after 48 h of incubation in either biofilm (Fig. 4; Table S4), indicating that native energy and substrate sources have been nearly depleted, or that all microorganisms capable of utilizing these under the applied experimental conditions became active within 48 h. The 48-h incubation time point was thus selected to further inspect the effects of different substrate amendments on the fraction and identity of the active biofilm populations.

Glucose, acetate, and thiosulfate amendments all resulted in a significant increase of the active bacterial fraction (as defined by positive signal with the EUBI-III probe, and a positive BONCAT signal) after 48 h of incubation (Fig. 4). The glucose amendment resulted in the highest total active cell biovolume in the Bizovac biofilms (58%) and the thiosulfate amendment had the greatest effect on the total active cell fraction in the Tuhelj biofilms (62.5%), while the activity stimulated by acetate and glucose amendments was much lower (Fig. 4; Table S4). Moreover, the activity stimulated by acetate and glucose amendment in Tuhelj biofilms was lower than the activity stimulated by the same substrates in Bizovac biofilms (Fig. 4; Table S4). Because *Gammaproteobacteria*- and *Chloroflexota*-related ASVs were the most abundant non-cyanobacterial ASVs in both biofilms (Fig. S2), the contributions of these taxa were selected for a more in-depth analysis of the observed responses to substrate amendment by BONCAT–CARD-FISH.

*Gammaproteobacteria* accounted for 22.8% of the initial population in Bizovac biofilms, including high relative abundances of the families *Rhodocyclaceae* (10.6%), *Hallothiobacillaceae* (4.9%), and *Hydrogenophilaceae* (1.8%), known to harbor thiosulfate oxidizers (Fig. S2). In the Tuhelj biofilms, *Gammaproteobacteria*-related ASVs accounted for 11.2% of the total microbial population, with most ASVs belonging to the families *Rhodocyclaceae* (3.6%), *Chromatimaceae* (1.1%), and *Beggiatoaceae* (1.0%) (Fig. S2). The fraction of active *Gammaproteobacteria* cells (defined as displaying a CARD-FISH signal with the Gam42a probe, and a positive BONCAT signal) also remained constant when comparing 24- and 48-h incubations without substrate amendment (Fig. 4). Under these conditions, ~25% of the gammaproteobacterial cells were active in the Bizovac biofilms, whereas only ~10% of the gammaproteobacterial population was active in Tuhelj samples (Table S4; Fig. 4). Glucose and thiosulfate amendments resulted in very similar responses of *Gammaproteobacteria* in Bizovac biofilms, with 56% and 61% of translationally active cells after 48 h of incubation, respectively (Fig. 4; Table S4). The acetate amendment had a negative effect on the activity of gammaproteobacterial cells in the Bizovac biofilms, and a BONCAT signal was detected in only 12.5% of the Gam42-positive cells after 48 h of incubation (Fig. 4; Table S4). In the Tuhelj biofilms, thiosulfate amendment resulted in a large increase of the active fraction of gammaproteobacterial cells, with 48% of translationally active Gam42a-positive cells after 48 h of incubation (Fig. 4; Table S4). The glucose amendment also resulted in increased activity of *Gammaproteobacteria* in the Tuhelj biofilms, with 36% of cells displaying activity after 48 h (Fig. 4; Table S4). As observed previously for the Bizovac biofilms, acetate amendment also led to a suppression of gammaproteobacterial activity in the Tuhelj biofilms. After 48 h of incubation with acetate, only 3.6% of gammaproteobacterial cells were translationally active (Fig. 4; Table S4).

In the Bizovac biofilms, *Chloroflexota* had a lower relative abundance than *Gammaproteobacteria*. Almost all ASVs affiliated to *Chloroflexota* (11.1%) belonged to the SBR1031 clade (4.4%) or the family *Anaerolineaceae* (6.1%) (Fig S2). In Tuhelj spring biofilm sample, *Chloroflexota*-related ASVs displayed a higher relative abundance then *Gammaprotobacteria* and accounted for the majority of the non-cyanobacterial microbial community (14.7%). Most ASVs in these samples were affiliated to the families *Chloroflexaceae* (8.9%) and *Anaerolineaceae* (2.9%) (Fig. S2). Less than 2% of *Chloroflexota* cells (defined as displaying a CARD-FISH signal with the CFX1223 probe, and a positive BONCAT signal) were active after 24 h, while 9% of cells were active after 48 h of incubation without substrate amendment in Bizovac biofilms (Table S4; Fig.4). In the Tuhelj biofilms, the activity of *Chloroflexota* cells in treatments without substrate amendment was much higher than in Bizovac biofilms, with 19% and 15% of active CFX1223-positive cells after 24 h and 48 h of incubation, respectively (Fig. 4; Table S4). Like the activity in the control treatment (no substrate amendment), the response of *Chloroflexota* populations in the Bizovac biofilms to glucose and thiosulfate was lower than the response of the gammaproteobacterial population. After 48 h of incubation in these substrates, 30% and 29% of the population were determined active, respectively (Fig. 4; Table S4). However, unlike *Gammaproteobacteria*, the *Chloroflexota* population in the Bizovac biofilms did respond with increased activity to acetate amendment, with 21% of active *Chloroflexota* cells after 48 h of incubation (Fig. 4; Table S4). In contrast, incubation with acetate in the Tuhelj biofilms resulted in lower activity of *Chloroflexota* compared with the control treatments. After 48 h of incubation with acetate amendment, only 9% of *Chloroflexota* cells were active (Fig. 4; Table S4). On the other hand, glucose amendments resulted in the highest increase in activity and percentage of active *Chloroflexota* cells in Tuhelj biofilms (47% after 48 h of incubation). Lastly, 37% of the *Cholorflexota* populations in the Tuhelj biofilms were active after 48 h of incubation in thiosulfate amended setups (Fig. 4; Table S4).

## Discussion

The microbial composition of hot spring biofilms has often been described to be mainly influenced by the elevated temperature in these environments (e.g., [7, 24, 28, 87]). However, a study of 925 hot springs in New Zealand showed that temperature has a significant effect on microbial beta diversity only at values above 80°C [88]. Although geothermal spring temperatures were lower in this study (max. 65°C), temperature, together with the sample origin, were the main determinants of biofilm microbial structure (Table S3). In contrast, no significant effect of sampling season or biofilm color on microbial community composition was observed. The most abundant microbial groups found in the sampled biofilms were consistent with the literature [89–96], with *Cyanobacteria* dominating the biofilms of all but one hot spring (Bizovac well), and *Chloroflexota*, *Pseudomonadota*, and *Bacteroidota* displaying a high relative abundance in all biofilms. The *Leptolyngbya*-related cyanobacteria, which dominated most of the biofilms we analyzed, also dominated the upper layer of alkaline microbial mats from the Garga hot spring [97], while the genus *Tychonema*, which we found in biofilms from the low-temperature well DB, was recently found abundant in biofilms from hydrothermal systems in Italy with temperatures moderately above ambient [98]. As expected, the microbial communities in biofilms were clearly distinct from the geothermal waters communities in the same springs. For example, Archaea were not abundant in biofilm sample (Fig. 3), while diverse crenarchaeal, micrarchaeal, and nanoarchaeal lineages dominated many of the water samples collected form the same springs [63]. The highest abundance of *Creanarchaeota* was found in the biofilm from the Topusko well (TEB) the sampling site with the highest temperature (64.8°C) in this dataset. Comparison of mats from a geothermal spring in Romania at different temperature ranges showed that the relative abundance of Archaea was low at 32°C (<0.5%), but increased significantly at 65°C (36%) [91], which is in line with our observations. Similarly, Alcaman et al. reported a temperature value of 58°C as the turnover point for phototrophs, with *Cyanobacteria* dominating the lower temperature range and *Chloroflexota* being more abundant in the 58–66°C range [93]. Three of our high-temperature sampling sites (Topusko well (TEB), Varazdin well (V), and Bizovac well (B)) displayed temperatures around this tipping point (Table S1), but only biofilm samples from Bizovac well were not dominated by *Cyanobacteria* and had high relative abundances of *Chloroflexota* (genera *Candidatus* Chlorotrix and *Chloroflexus*; Fig. S1). All other high- and moderate-temperature sampling sites were in fact dominated by *Cyanobacteria* (Fig. 3b), confirming that temperature is not the only driver for the previously observed community switch among primary producers. Also, thiotrophs detected in water samples from the same sites (e.g., *Sulfuricurvum* and *Sulfurimonas* among the *Camplyobacterota*, and *Thiobacillus* among the *Gammaproteobacteria*, [63]) differed from the dominant genera in our biofilm samples (*Sulfurovum*, *Thiofaba*, *Thiotrix*, *Hydrogenophillus*), all previously reported to occur in hot spring biofilms from other sites with similar temperatures (e.g., [99]).

Based on the differences in microbial community composition and spring geochemistry observed during seasonal sampling, fresh biofilms were collected from the Bizovac well and the Tuhelj spring, and subjected to a series of incubation experiments to investigate the organo- and lihtotrophic activity of the non-photosynthetic community members. The majority of hot spring biofilms we analyzed, including those from Tuhelj spring, were dominated by *Cyanobacteria*, the dominant photosynthetic primary producers in these systems. Thus, *Cyanobacteria* are likely a major source of organic carbon substrates, sustaining growth and activity of chemoorganotrophic microorganisms residing in the hot spring biofilms. However, as the hot spring biofilms grow at a redox interface, a fraction of the microbial community might also be adapted to derive energy from reduced inorganic substrates via chemolithotrophic metabolisms, as evidenced, for example, by the presence of various and diverse thiotrophs in most biofilm samples (Fig. S1). Therefore, thiosulfate was selected as substrate to test chemolithotrophic activity related to the geochemical gradients the biofilms reside on. Glucose and acetate were selected as carbon substrates to simulate activity of chemoorganotrophs, in situ supplied with organic compounds via photosynthetic primary production. In biofilms recovered from both sources, the thiotrophs putatively able to oxidize the thiosulfate provided as substrate were predominantly affiliated with various gammaproteobacterial lineages.

The Bizovac biofilm included relatively more ASVs affiliated with putative thiotrophs and *Gammaproteobacteria* in general compared to *Cyanobacteria*-dominated biofilms from the Tuhelj spring (Fig. S2). ASVs related to *Chromatiaceae* and *Hallothiobacillaceae* were identified as putative thiotrophs in the Bizovac sample, while *Chromatiaceae* and *Beggiatoaceae* ASVs were found in the biofilms from the Tuhelj spring (Fig. S2; [100]). Other abundant gammaproteobacterial families in both samples were *Rhodocyclaceae* and *Hydrogenophilaceae* (Fig. S2). The family *Rhodocyclaceae* is metabolically diverse and consists of photoautotrophic, heterotrophic, and organotrophic members. This family has also been found abundantly in sulfidic hot springs in northern Baikal [101] and in microbial mats of hot springs in Eritrea [102]. The most abundant ASVs of the family *Rhodocyclaceae* detected in the Bizovac biofilm belonged to the genus *Azoarcus* (8%). Although primarily classified as denitrifying chemolithoheterotrophs, numerous studies reported *Azoarcus* to be core denitrifying desulfurizing bacteria in bioreactor experiments under mixotrophic conditions [103–107]. The only currently known sulfur-oxidizing denitrifying species, *Azoarcus taiwanensis*, was isolated from a hot spring biofilm in Taiwan [108]. Members of the family *Hydrogenophilaceae* are thermophilic heterotrophs and autotrophs that can use hydrogen as an electron donor [109]. A new species of this family was recently isolated from microbial mats of hot springs in Japan that can grow with thiosulfate and elemental sulfur under aerobic conditions and is unable to utilize hydrogen [110]. Almost all *Hallothiobacilaceae* ASVs in the Bizovac sample belonged to the genus *Thiofaba* (4.8%; Fig. S1). The type strain of this genus, *Thiofaba teidiphila*, was isolated from the biofilm of a hot spring in Japan and characterized as an obligate chemolithoautotrophic bacterium that utilizes thiosulfate, elemental sulfur, sulfide, and tetrathionate as electron donors [111]. Furthermore, a larger fraction of the microbial community in Bizovac biofilms was related to *Desulfobacterota*—a phylum harboring various microorganisms involved mainly in reductive, but also oxidative sulfur cycling [100]. Also, appreciable amounts of sulfide were detectable in situ in the water samples from Bizovac well, but not in waters of the Tuhelj spring (Table S1), indicating sulfide oxidation, but also S-disproportionation [112]. Hence, we hypothesized that either a larger fraction of the microbial community in the Bizovac biofilms would be capable of chemolithotrophic activity utilizing thiosulfate as source of reducing equivalents, or that the observed changes in the activity of the non-thiotrophic fraction, despite the relatively short incubation time, were the results of cross-feeding interactions in which the activity of the putative thiotrophs led to the accumulation of hydrogen and small organic molecules. Contrary to our hypothesis, the overall increase in the total active cell fraction and, in particular, the active gammaproteobacterial cell fraction was more pronounced in the Tuhelj biofilms in incubations amended with thiosulfate (Fig. 4), in which ASVs related to known thiotrophs were relatively less abundant (Fig. S2). Because the community in Bizovac biofilms was dominated by chemolithoheterotrophic *Rhodocyclaceae*, it is possible that the availability of suitable organic carbon sources limited the metabolic activity of thiotrophs in our experiments.

In both biofilm samples, the response to glucose amendments in the gammaproteobacterial cell fraction was in the same range of activity increase as in the thiosulfate amended incubations (Fig. 4). These results are in line with chemoorganoheterotrophy and mixotrophy generally expected for many of the community members detected here. The class *Gammaproteobacteria* contains the largest diversity of obligate and generalist hydrocarbonoclastic bacteria [113], many of which are known for their implication in acetate assimilation [114, 115]. However, contrary to our expectation, incubation with acetate resulted in decreased fractions of active gammaproteobacterial populations in both biofilms. Compared to glucose, acetate is a low-energy carbon source and has been shown to have an inhibitory effect on some microorganisms, reducing their growth rate [116]. However, as the overall response of the microbial community to acetate amendment was increased activity, the used acetate concentration does not appear to be generally inhibitory.

In both biofilms, the increase in the active cell fraction of *Chloroflexota* in incubations with thiosulfate was almost equivalent to the activity increase in glucose treatments (Fig. 4). This result was rather surprising, as the majority of *Chloroflexota* ASVs in both samples were not related to known thiotrophs. Almost all *Chlorofexota* ASVs in the Bizovac sample belonged to the clade SBR1031 and the family *Anaerolineaceae*. These were previously found to occur in microbial mats at temperatures of 25–60°C in hot springs of the Tibetan Plateau [117], Costa Rica [118], and Saudi Arabia [119]. Most members of the *Anaerolineaceae* are known to be mesophilic or thermophilic chemoheterotrophs that grow mainly under anaerobic conditions and metabolize sugars fermentatively [120]. In addition to fermentative sugar metabolism, SBR1031 members also encode key genes for acetogenic dehydrogenation [121]. Thus, the stimulation of *Chloroflexota* by thiosulfate amendments observed here was unexpected. It is possible that either members of *Chloroflexota* with yet undescribed metabolism are present in the biofilm samples, or that despite the relatively short incubation time, we are observing the results of microbial interactions in which compounds produced and released by thiosulfate oxidizers, such as the thiotrophic gammaproteobacterial community, actually stimulated *Chloroflexota*. On the other hand, *Chloroflexota* ASVs from the initial Tuhelj biofilms, apart from the *Anaerolineaceae* family, mostly belonged to the *Chloroflexaceae* family, known to grow photoautotrophically, suggesting an ecological role as primary producers in hot spring environments [122], but also previously shown to utilize acetate [123]. Still, the acetate amendment in this biofilm resulted in slightly reduced activity of *Chloroflexota*, contrary to our expectations.

## Conclusion

In this study, we for the first time explored the microbial community composition of biofilms from geothermal springs and wells in northern and north-eastern Croatia. We showed that biofilm microbial communities display a stable and site-specific composition over three sampling seasons, and determined that temperature and spring source had the greatest influence on biofilm community composition, while all other tested physicochemical factors showed only minor influence. Based on this biofilm diversity screening, two geographically distant and geochemically distinct hot springs, hosting biofilms with very dissimilar microbial communities, were selected for further activity-based analyses. The here utilized method, BONCAT–CARD-FISH, has proven to be a rapid and comparatively simple way to investigate the ecophysiology of geothermal biofilm communities, allowing metabolic and taxonomic screening not constrained by cultivation limitations. However, although we demonstrated chemoorganotrophic and thiosulfate-dependent chemolithotrophic activity in both biofilms, BONCAT–CARD-FISH cannot directly link substrate uptake to microbial activity due to likely secondary interactions of microorganisms. Many results obtained in the performed activity measurements contradicted our hypothesis on dominant metabolism and likely involved biofilm members that we had derived from hot spring geochemistry and 16S rRNA gene amplicon community profiling. This outcome emphasizes the necessity for further physiology- and activity-targeted experimentation to fully understand microbial interactions and metabolism, even within the relatively well-defined habitat of hot spring biofilms.

### Supplementary Information

ESM 1 (DOCX 813 kb)

ESM 2 (XLSX 29 kb)

## Data Availability

16S rRNA gene amplicon sequencing data have been deposited in SRA (the Sequence Read Archive) under the BioProject accession number PRJNA889237.
