# Supplementary material for: Microbial Diversity and Activity of Biofilms from Geothermal Springs in Croatia
Source: Microb Ecol. 2023 May 20;86(4):2305–19. doi: 10.1007/s00248-023-02239-1 (PMC10640420; doi:10.1007/s00248-023-02239-1)
Supplement: Supplementary file 1 — (DOCX 813 kb) [file 248_2023_2239_MOESM1_ESM.docx]

**Supplementary material**

*On site measurements*

After sampling, a multiparameter probe (WTW) was used to measure the following physical parameters *in situ*: electrical conductivity (EC) with an accuracy of ± 1 μS cm^-1^, dissolved oxygen in water (O_2_) with an accuracy of ± 0.1 mg L^-1^, pH with an accuracy of ± 0.01, and water temperature (°C) with an accuracy of ± 0.1 ºC. Concentration of hydrogen sulfide (H_2_S) in water samples were determined by a colorimetric method using a HACH DR3900 spectrometer.

*Measurements in the laboratory*

In the Hydrochemical laboratory of the Department of Hydrogeology and Engineering Geology of the Croatian Geological Institute, water samples were analyzed the same evening after returning from the field. The concentrations of cations (sodium, potassium, calcium, ammonium and magnesium) and anions (fluoride, bromide, chloride, nitrate, sulfate and iodine) were measured by an ion chromatographic method using the Dionex ICS-6000 DC instrument. Alkalinity was measured by a titration method using 1.6 N H_2_SO_4_ and phenolphthalein and bromocresol green methyl red indicators. SiO_2_ concentrations were measured by a colorimetric method using a HACH DR3900 spectrometer. The analytical precision of the cation and anion measurements, expressed as ion balance error (IBE), was calculated based on the sum of ions expressed in meq L^-1^ using the program NETPATH-WIN (El-Kadi et al., 2010). The IBE values are within the limits of < ± 5% (Mandel & Shiftan, 1980; Domenico & Schwartz, 1990), indicating high reliability of the analyses. Dissolved organic and inorganic carbon (DOC and DIC) was measured using a QbD1200 liquid carbon analyzer from HACH. The analyzer uses H_3_PO_4_ to acidify the sample, converting dissolved inorganic carbon to CO_2_. Subsequently, in the presence of UV radiation and a strong oxidant, (NH_4_)_2_S_2_O_8_, the organic carbon is oxidized to carbon dioxide and the total CO_2_ is measured with a non-dispersive infrared detector. The instrument detects the CO_2_ signal and displays it as a curve. The area under the curve represents the DOC values. Before analysis, the sample is filtered through a 0.2 μm filter.

References:

EL-Kadi A. I., Plummer L. N., Aggarwa L P. 2010: NETPATH-WIN: An Interactive User Version of the Mass-Balance Model, NETPATH. Ground Water, 49/4, 593-599.

Domenico PA, Schwartz FW. Physical and Chemical Hydrogeology. New York. John Willey and Sons;1990.

Mandel S, Shiftan ZL. Groundwater Resources Investigation and Development. New York. Academic Press;1980.


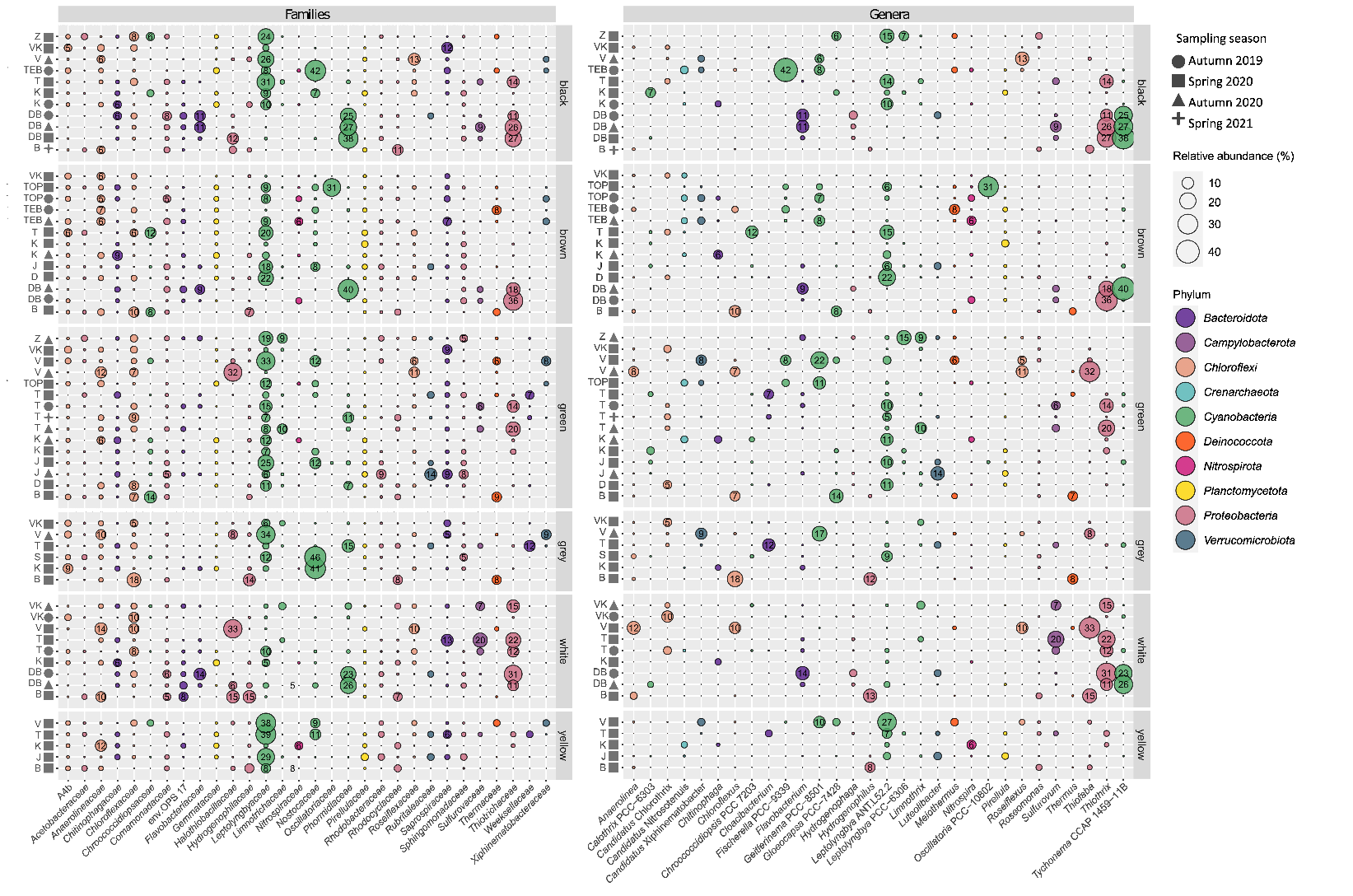


**Fig. S1** Microbial community composition at the family and genus level. Bubble plot of the average relative abundance (circle size) of 30 most abundant families and genera, colored according to their phylum affiliation. Samples are grouped by the color of the biofilm (sub)samples. Sampling locations are indicated by sampling site abbreviation, while shapes represent the season of sampling.


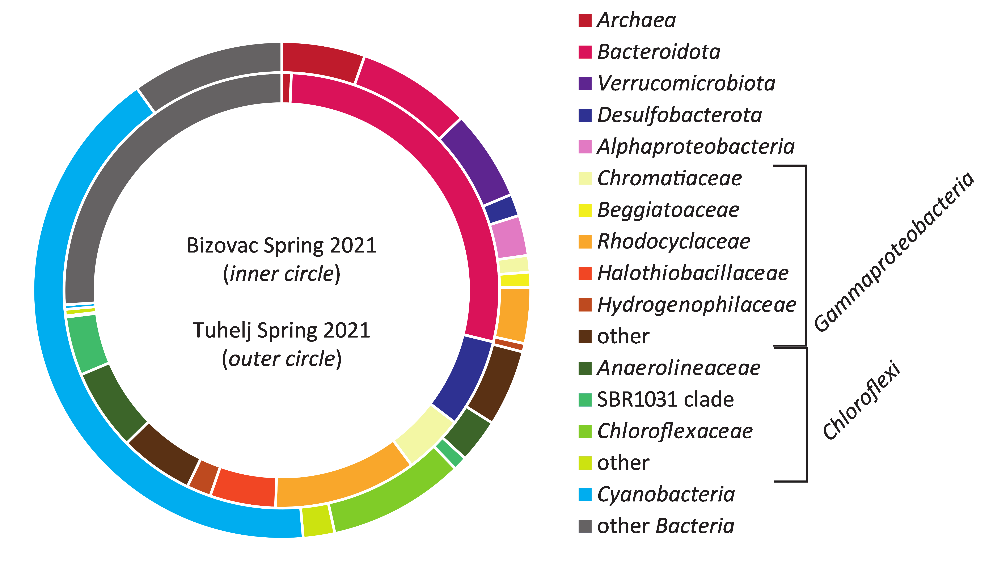


**Fig. S2** Family-level microbial community composition of biofilms from the Bizovac well and the Tuhelj spring, collected in spring 2021, on which incubation experiments were performed
